# Indirect calorimetry is the gold standard to assess REE in ICU patients: some limitations to consider

**DOI:** 10.1186/s13054-021-03817-w

**Published:** 2021-11-25

**Authors:** Patrick M. Honore, Sebastien Redant, Thierry Preseau, Keitiane Kaefer, Leonel Barreto Gutierrez, Sami Anane, Rachid Attou, Andrea Gallerani, David De Bels

**Affiliations:** 1 ICU Dept, Centre Hospitalier Universitaire Brugmann-Brugmann University Hospital, Place Van Gehuchtenplein, 4, 1020 Brussels, Belgium; 2 ED Dept, Centre Hospitalier Universitaire Brugmann, Brussels, Belgium

**Keywords:** Indirect calorimetry, REE, CRRT, ECMO

In their metanalysis, Duan et al. address the role of indirect calorimetry (IC) in nutritional therapy in critically ill patients [1].Their findings support using IC rather than predictive equations as the gold standard to assess resting energy expenditure (REE) [1]. Previous studies have demonstrated the low accuracy of various REE predictive equations based on weight, height, age, gender, etc. [1]. Despite adjustments according to patient population and other modifying factors, REE discrepancies remain, (with variations up to 60%) [1]. IC allows for the measurement of VO2 and VCO2 through the ventilator and is the gold standard method for measuring REE in ICU, when ideal test conditions are implemented [1]. Both the European (ESPEN) and American (ASPEN) clinical practice guidelines recommend the use of IC to measure REE [2]. While supporting the use of IC in some settings, we wish to point out a number of limitations, particularly when patients are undergoing continuous renal replacement therapy (CRRT) [3] and extracorporeal membrane oxygenation (ECMO) [4, 5]. Estimating REE using IC in CRRT patients is less reliable for several reasons [3]. First, CO2 from bicarbonate-based dialysate can pass the filter and circulate in the form of dissolved CO2, bicarbonate, or carbamino compounds in red blood cells or plasma [3]. Though a quantity of CO2 may be removed in the effluent [3], a recent study showed that CO2 removal by CRRT led to a minimal change of 3% of measured EE [3] Second, patients may experience heat loss up to 1000 kcal during CRRT, resulting in increased metabolism and REE [3]. Third, dialysate compositions and citrate also contribute to caloric uptake [3]. For all these reasons, IC remains less reliable during CRRT and more research should shed light [3]. This is also true for IC performed in patients on ECMO, unless a mathematical correction is applied [3]. It is important that clinicians are aware not only of the indications for IC, but also its limitations. [3]. Other technical limitations of IC in ICU are discussed in a comprehensive review to which readers should refer [5].

## Data Availability

Not applicable.

## Abbreviations

IC: Indirect calorimetry
REE: Resting energy expenditure
ICU: Intensive care unit
ESPEN: European society for parenteral and enteral nutrition
ASPEN: American society for parenteral and enteral nutrition
CRRT: Continuous renal replacement therapy
ECMO: Extracorporeal membrane oxygenation
CRRT: Continuous renal replacement therapy
ECMO: Extracorporeal Membrane Oxygenation
IC: Indirect calorimetry
REE: Resting energy expenditure
RCTs: Randomized controlled trials
