# Supplementary material for: Indirect calorimetry is the gold standard to assess REE in ICU patients: some limitations to consider
Source: Crit Care. 2021 Nov 25;25:406. doi: 10.1186/s13054-021-03817-w (PMC8613971; doi:10.1186/s13054-021-03817-w)
Supplement: Related texts

# Reply: Response to the letter to the editor: “Using indirect calorimetry in place of fixed energy prescription was feasible and energy targets were more closely met: do not forget an important limitation”

**Authors:** Hui-Bin Huang, Jing-Yi Duan, Yuan Xu, Hua Zhou

We thank Professor Honore et al. for their interest in our meta-analysis [1]. Our study identified 8 RCTs and demonstrated that compared with predictive equations, indirect calorimetry (IC) guided energy delivery can significantly reduce short-term mortality in critically ill patients without affecting other important clinical outcomes such as hospital stay and mechanical ventilation.

We fully agree with what Professor Honore and colleagues pointed out that clinicians need to understand not only indications of IC but its limitations [6]. Especially, Professor Honore highlighted that the use of IC might be unreliable in patients undergoing continuous renal replacement therapy (CRRT) and extracorporeal membrane oxygenation (ECMO). Indeed, although all included RCTs in our meta-analysis provided detailed IC indications, only two trials had excluded patients receiving CRRT, and no trials reported having excluded patients with ECMO [1].

Cardiopulmonary bypass can remove part of VCO2, which is considered the most important technical factor affecting the reliability of IC measurement [6]. However, this does not mean that patients who receive CRRT or ECMO are bystanders of the IC technique. In a monocentric, controlled, prospective, observational pilot study, Wollersheim, and colleagues proposed measuring the reliability of energy expenditure (EE) in extracorporeal lung support patients by calculating the O2 uptake and the CO2 elimination by the ECLS membrane, then using sum O2 uptake and CO2 elimination in the equation of Weir to calculate EE [7].

Although CRRT may be more complicated due to factors such as replacement fluid, Jonckheer et al. proposed a simple blood gas analysis based on the circuit to quantify CO2 that was removal during continuous venovenous hemofiltration. In their study, the CO2 content in ultrafiltration is available by blood gas analysis, and the CO2 content is converted from mmoL to mL with the help of the ideal gas law (pV = nRT). The authors performed IC in 4 different states (baseline, high dose, baseline with NaCl predilution and without CVVH) and found that CO2 removal by CVVH led to a change in REE of only 3%, which makes a correction factor unnecessary in this setting [8]. Although the authors believed that citrate might be the only factor that significantly changes metabolism out of all the potential metabolic influences during CVVH, they also admitted that this required further research [8].
